# Outcome and prognostic factors of desmoplastic medulloblastoma treated within a multidisciplinary treatment concept

**DOI:** 10.1186/1471-2407-10-450

**Published:** 2010-08-23

**Authors:** Stefan Rieken, Timo Gaiser, Angela Mohr, Thomas Welzel, Olaf Witt, Andreas E Kulozik, Wolfgang Wick, Jürgen Debus, Stephanie E Combs

**Affiliations:** 1 University Hospital of Heidelberg, Department of Radiation Oncology, Im Neuenheimer Feld 400, 69120 Heidelberg, Germany; 2 University Hospital of Heidelberg, Department of Neuropathology, Im Neuenheimer Feld 220/221, 69120 Heidelberg, Germany; 3 University Hospital of Heidelberg, Department of Pediatric Oncology, Hematology, Immunology, and Pneumonology, Im Neuenheimer Feld 430, 69120 Heidelberg, Germany; 4 University Hospital of Heidelberg, Department of Neurooncology, Im Neuenheimer Feld 400, 69120 Heidelberg, Germany

## Abstract

**Background:**

Desmoplasia in medulloblastoma is often diagnosed in adult patients and was repeatedly associated with improved results. Today, all medulloblastoma patients receive intensive multimodal treatment including surgery, radiotherapy and chemotherapy. This study was set up to investigate treatment outcome and prognostic factors after radiation therapy in patients with desmoplastic medulloblastomas.

**Methods:**

Twenty patients treated for desmoplastic medulloblastoma in the Department of Radiation Oncology at the University of Heidelberg between 1984 and 2007 were included. Data were collected retrospectively. Tumor resection was performed in all patients. All patients underwent postsurgical radiotherapy (RT). Two patients underwent whole brain radiotherapy (WBRT), and 18 patients received craniospinal irradiation (CSI). In all patients, an additional boost was delivered to the posterior fossa. The median dose to the whole brain and the craniospinal axis was 35.2 Gray (Gy), and 54.4 Gy to the posterior fossa. Fourteen patients received chemotherapy, including seven who were treated with combined radiochemotherapy and twelve who received adjuvant chemotherapy. Statistical analysis was performed using the log-rank test and the Kaplan-Meier method.

**Results:**

Median follow-up was 59 months. Overall (OS), local (LPFS) and distant progression-free survival (DPFS) was 80%, 71.2%, and 83.3% at 60 months. Patients who suffered from local or distant relapses had significantly worse outcome. Five patients died from recurrent medulloblastoma. Treatment-associated toxicity was acceptable.

**Conclusions:**

Multimodal approaches with surgical resection followed by chemoirradiation achieved high response rates with long OS in desmoplastic medulloblastoma patients. Staging parameters expected to predict for poor prognosis did not significantly influence outcome. However, success of any first line regimen had strong impact on disease control, and remission was achieved in no patient with relapsing disease. Multimodal concepts must be evaluated in further clinical trials.

## Background

Medulloblastoma is the most common and most malignant embryonal tumor of the pediatric central nervous system (CNS). It is believed to originate from neuroepithelial cells, lining the roof of the fourth ventricle [1]. Different histological subtypes of varying neuronal differentiation have been described, and among these desmoplastic medulloblastoma, known for its nodular architecture, has been discussed controversially with respect to its impact on therapy response and outcome.

Most medulloblastoma patients are diagnosed between five and ten years of age, accounting for 25% of all pediatric brain tumors [2]. Medulloblastomas occur rarely in adolescents and adults, however about 1% of all non-pediatric brain tumors are identified as primitive neuroectodermal tumors (PNET), among which medulloblastoma is the major subgroup [3,4]. In this population, a clear distinction must be made between infratentorial and supratentorial medulloblastomas, in the latter location also referred to as PNET, which despite histological resemblance are associated with poorer outcome due to their rapidly invasive growth [5]. Early locoregional infiltration has been shown to compromise local control rates and predispose for systemic spread of disease via the neuraxis in case of supratentorial location [6].

Lateral tumor location, desmoplastic histology variant, and recurrence of disease after longer time periods are only some of the main distinguishing features between medulloblastoma in adults and in children [7].

Continuously evolving therapeutical strategies including adjuvant RT and extensive concomitant and adjuvant chemotherapy have helped to substantially improve survival rates after surgical resection in children [8]. In adults however, no optimal standard treatment regimen has been developed, most likely due to small patient numbers. This has led to numerous reports on individual treatment strategies for non-pediatric patients, in which either combination or adjuvant addition of chemotherapy was abandoned or dosage and fractionation scheme of irradiation were altered. Different histological patterns, sites of tumor location and recurrence among non-pediatric patients are poorly understood. Their impact on response and outcome has been addressed several times, yet remains unclear until now [9]. In the past, most centers have treated adult patients in analogy to pediatric multidisciplinary treatment protocols including extensive neurosurgical resection, CSI and chemotherapy. However, it is known that toxicitiy profiles may vary substantially between children and adults [10]. Therefore, multidisciplinary treatment approaches are currently being investigated in an observational study conducted by the Neurooncology Group of the German Cancer Society which recently started accrual (NOA-07 Trial, http://www.neuroonkologie.de).

Progression-free survival rates (PFS) reported in the literature vary between treatment strategies and centers and are reported to be between 35% and 95% at five years; a number of risk factors have been identified to potentially influence treatment response and allow for outcome predictions [11,12]. Risk-adapted therapy approaches, however, require clear identification of prognostic factors, which among others include extent of surgery, initial disease dissemination, locoregional infiltration and age at primary diagnosis [13].

The desmoplastic histology variant was repeatedly described to be indicative of low risk but its significance as a potential predictor of good response still remains unclear [11,14,15]. In children younger than three years in whom no adjuvant radiotherapy is administered, desmoplastic histology was associated with PFS as high as 85% after ten years, opposed to classic histology with 34% [16], questioning the necessity for radiation in desmoplastic histology variant. Within a group of adult medulloblastoma, higher percentages of desmoplasia must be expected than in previous, mostly pediatric studies, ranging from 25% to 40% [7,17]. Since the German Neurooncology Group (NOA) has initiated a new study focusing upon medulloblastomas in adults (NOA-07 Trial), once again attention must be paid to the impact of desmoplasia on therapy outcome, since this subtype must be expected more frequently in the adult population. Prognostic factors within the group of desmoplastic medulloblastoma patients have not been addressed to date. Recently, few molecular DNA-based parameters such as 6q-deletion were identified to predict for good prognosis in medulloblastoma in general including desmoplastic histology [18]. However, no stratification of these patients into risk adapted therapy groups has been established. For the establishment of high and low risk therapy groups, several factors of potential threat can be considered, some of which are considered also in classical medulloblastoma [9].

In the present analysis, we evaluate outcome in the rare group of patients with histologically confirmed desmoplastic medulloblastomas treated within multidisciplinary concepts.

## Methods

This project was performed in accordance with institutional ethical policies. No ethical approval was necessary, because all data were collected retrospectively from an institutional patient database and tumor registry. They were collected anonymously using a uniform retrieval code ("desm. mb") for data acquisition.

Between 1984 and 2007, 20 patients with histologically confirmed desmoplastic medulloblastomas were treated at the Department of Radiation Oncology in Heidelberg, Germany. Median follow-up time was 58.5 months (range 5 - 254 months). Two patients were lost to further follow-up after 5 and 60 months, respectively.

### Patient characteristics

Thirteen patients were male (65%), and 7 were female (35%). Median age at primary diagnosis was 24 years (range 3-50 years). The youngest patient was three years old. Seven patients were 18 years of age or younger (35%), while 13 patients (65%) were older than 18 years, with 11 patients being older than 21 years (55%).

### Surgery and histopathological findings

In order to relieve ventricle obstruction, to obtain tissue for neuropathological analysis and to reduce tumor volume by complete or partial tumor resection, all patients underwent neurosurgical resection before RT. In 13 patients a complete tumor resection was achieved (65%), while in 7 patients a subtotal tumor resection was performed (35%). Extent of resection was defined on the basis of surgical reports and/or postoperative imaging, which was performed in ten patients with six of them receiving MRI and four of them receiving CT examinations using contrast agents. Ten patients did not undergo postoperative imaging leaving the assessment of resection extent to the surgeon. Tumor resection was declared incomplete in 3 of 7 patients by the operating neurosurgeon due to clear infiltration of critical non-resectable structures. They had macroscopic residual tumor, which in most cases infiltrated the brainstem via the fourth ventricle. In 4 further incomplete tumor resections, cross sectional imaging identified contrast-enhanced residual tumor tissue. Sixteen patients had lateral tumor location (80%), whereas 4 patients (20%) presented with midline tumors. In all patients complete staging of the craniospinal axis was performed using MR-imaging as well as examination of the cerebrospinal fluid (CSF).

In four patients intracranial or spinal dissemination was present at primary diagnosis (20%). During surgery, infiltration of the fourth ventricle was detected in 9 patients (45%). Out of these, a complete tumor resection was possible in 4 patients (44%). Eleven patients did not show direct affection of the fourth ventricle, and in this subgroup a complete resection was achieved in 9 patients (81.82%).

**Figure 1:**
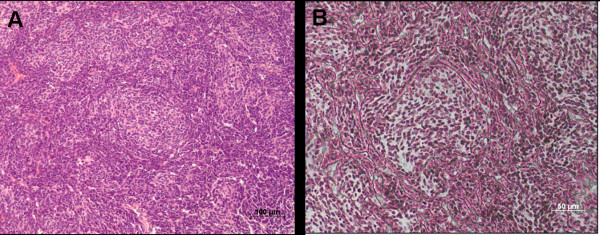
**Neuropathological diagnosis of desmoplastic medulloblastoma**. **A**: Rarefied nodules of reduced cellularity surrounded by densely packed hyperchromatic cells (100× magnification; HE-staining). **B**: Dense intercellular reticulin and nodular reticulin-free zones (200× magnification; TP-staining).

Neuropathological diagnosis was desmoplastic medulloblastoma in all patients according to the current classification criteria of the World Health Organization (WHO) for medulloblastoma [19]. Based on the features in hematoxyline/eosine and reticulin stained sections, desmoplastic medulloblastoma was diagnosed when having a biphasic architecture that consisted of regions with dense intercellular reticulin and nodular reticulin-free zones, within which tumor cells show a neurocytic phenotype (Figure 1).

### Radiotherapy

Postoperative RT was conducted as CSI and a subsequent boost to the posterior fossa in all but two patients. CSI was delivered with a median total dose of 35.2 Gy (range 23.4 - 36 Gy), and the boost to the posterior fossa was applied up to a total median dose of 54.4 Gy (range 48 - 68 Gy). Median single doses were 1.63 Gy for CSI and 1.8 Gy for boost irradiation, and ranged between 1 and 2 Gy. Boosts were applied with conventional X-ray-based techniques in 11 patients, while in 9 patients the boost was delivered using 3D-treatment planning based on CT- and MR-imaging. Treatment was delivered using 4 isocentric non-coplanar irregularly shaped fields formed by a multileaf collimator in individual mask fixation.

Radiation was delivered with 60 cobalt units in three patients, who were treated before 1991, and with linear accelerators in 17 patients. In one patient, irradiated with CSI and boost to the posterior fossa, helical intensity modulated radiotherapy (IMRT) using the tomotherapy technique was employed [20].

Two patients received whole brain radiotherapy (WBRT) with doses of 36 Gy and 40 Gy, respectively, followed by boosts to the posterior fossa with an additional 18 Gy and 8 Gy, respectively. One of these two patients required addition of local spinal RT for metastasis 42 months after WBRT.

### Chemotherapy

In one patient, before initiation of RT chemotherapy consisting of procarbazine, vincristine and methotrexate was administered. In seven patients RT was combined with chemotherapy, consisting of weekly infusions with vincristine. Twelve patients were treated with adjuvant chemotherapy after radiation with nine of them being enclosed within or treated in analogy to multimodal protocols conducted by the German Society for Pediatric Hematology and Oncology (GPOH, HIT-Studies). One patient received a chemotherapy consisting of vincristine and lomustine after completion of radiotherapy. Adjuvant chemotherapy with alternating methotrexate and a combination of methylprednisolone, vincristine, cytarabin, cisplatin, dacarbacine, lomustine, procarbacine and hydroxyurea ("8 in 1") was administered in one patient, who after suffering from local tumor recurrence was treated with various non-standard chemotherapy regimens as individual salvage concepts. Another patient without chemotherapy during first line treatment received two cycles of carboplatin and etoposide after locally relapsing disease. One further patient, who had received adjuvant post-irradiation procarbazine, vincristine, and methotrexate, was treated with "8 in 1" after developing local disease recurrence. One patient insisted to have received adjuvant chemotherapy of unknown regimen.

### Statistical analysis

Event-free survival was calculated from initial diagnosis until first relapse at any site or death from any cause. Overall, local and distant progression-free survivals were calculated in months from the date of surgery until the first diagnosis of recurrent disease. Overall survival (OS) was calculated from the date of primary diagnosis until the last date of follow up or death of the patient. Local and distant progression-free survival rates (LPFS and DPFS) were calculated from the date of surgery until the first imaging diagnosis of tumor relapse and were displayed with the Kaplan-Meier method. Results are displayed in% ± standard error of the mean (SEM). Survival curves were compared between groups by the log-rank test. All statistical analyses were performed with a software tool, "Statistika 6.1".

## Results

### Toxicity

Adjuvant RT was well tolerated and completed by all patients within the planned time intervals. Acute radiogenic toxicity was relatively mild. Most patients experienced temporary tiredness, and reported tolerable nausea and transient alopecia. Treatment-refractory nausea with vomiting (n = 2; 10%) and severe fatigue syndrome (n = 4; 20%) were rarely diagnosed.

More severe acute toxicity was related to the addition of chemotherapy. In five patients (25%), four of whom received chemotherapy, temporary leukocytopenia was observed and required postponing of further chemotherapy in three patients (15%). Only one patient, who did not receive chemotherapy, developed leukocytopenia during CSI. In this patient, boost irradiation was initiated before completion of CSI, and CSI was continued after boost application when blood values had recovered. During adjuvant chemotherapy, two patients reported severe abdominal pain and obstipation that was diagnosed as subileus with spontaneous remission in both cases (10%). Acute skin-related toxicity was observed in two patients who suffered from retroauricular ulcerations (10%).

Chronic toxicity comprised peripheral neuropathy in six patients, all of whom had been treated with concomitant or adjuvant vincristine (30%). One patient, who had received cisplatin in an adjuvant setting developed massive ototoxicity and finally underwent cochlea implant 16 years after tumor resection. Another patient, whose adjuvant chemotherapy was changed from cisplatin to carboplatin due to acute tinnitus after only one cisplatin administration, still was diagnosed with chronic high pitch deafness.

Mental development was impaired in three patients (15%), who had received RT without chemotherapy at the age of 3, 7, and 11 years, respectively, however successfully attended schools for mentally handicapped children. One female patient went through a phase of severe post-treatment depression that was related to transient infertility. Hypothalamic-pituitary dysfunction was ruled out in this case, yet detected in three other pediatric patients (15%) and led to hypothyroidism, pubertas tarda and retarded growth that required hormone substitution.

One patient was diagnosed with having Li-Fraumeni syndrome (LFS) several years after being treated for medulloblastoma and developed further LFS-spectrum tumors including intraabdominal atypical teratoid rhabdoid tumor and myelodysplastic syndrome. Radiation-associated osteosarcoma of the skull base occurred seven years after RT in one patient, who had been treated at the age of seven years. This tumor was incompletely resected and additionally irradiated with single doses of 1.8 Gy delivering a total dose of 36 Gy. After six years, this patient is alive and disease-free.

### Survival

Thirteen patients are alive at the time of this analysis. Follow-up examinations including neuroimaging were conducted for a median of 59 months, during which 7 patients suffered from relapse of disease (35%). Five of seven patients with relapsing disease died from medulloblastoma (71.4%). Both patients with recurrent disease, for whom no dates of death are known, had developed spinal metastases and were lost to further follow-up briefly after diagnosis of relapse, indicating probable death. None of 13 patients found disease-free after initial treatment died within the follow-up time.

**Figure 2:**
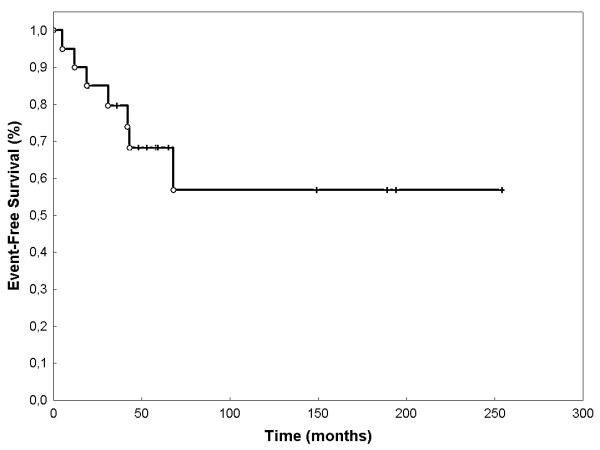
**Event-free Survival**: EFS was 90.0% at 12 months, 85.0% at 24 months, 68.3% at 60 months and 54.9% at 120 months (Kaplan-Meier, n = 20).

**Figure 3:**
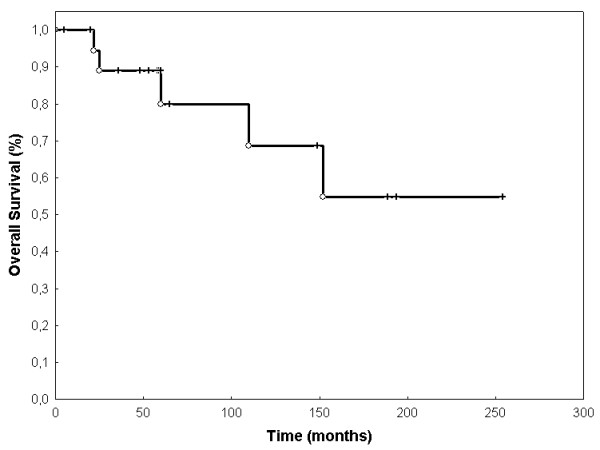
**Overall survival**: OS was 94,4% at 12 months, 88,9% at 24 months, 80% at 60 months and 54,9% at 120 months (Kaplan-Meier, n = 20).

**Figure 4:**
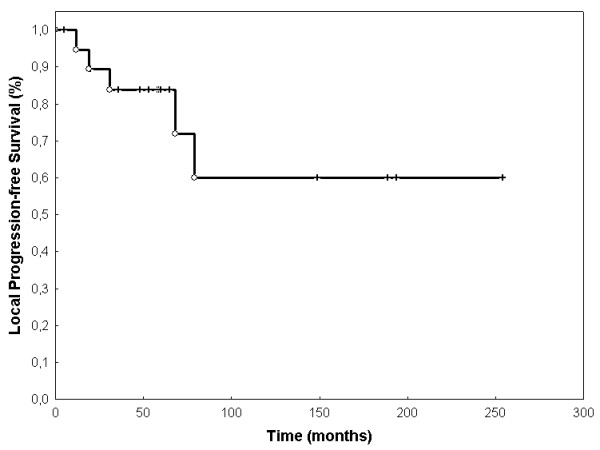
**Local progression-free survival**. LPFS was 94,7% at 12 months, 83,9% at 24 months, 71,2% at 60 months, and 59,9% at 120 months (Kaplan-Meier, n = 20).

**Figure 5:**
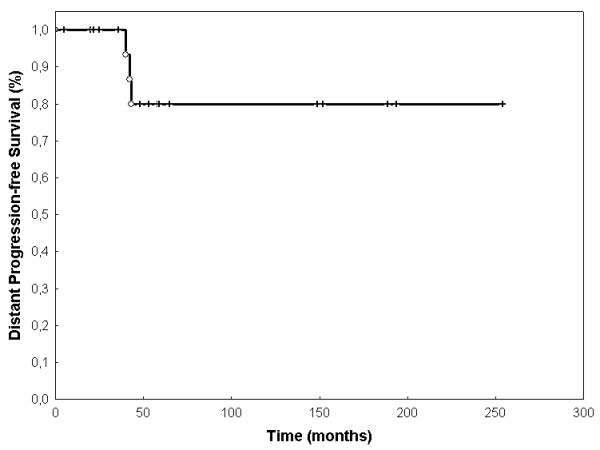
**Distant progression-free survival**. DPFS was 93,3% after 36 months and 83,3% after 60 months with no further recurrences after 43 months (Kaplan-Meier, n = 20).

Event-free survival (EFS) was 68.3% (SEM 0.107) after 60 months (figure 2). OS was 80% (SEM 0.107) at 60 months (Figure 3). LPFS was 83.8% (SEM 0.085) at 60 months (Figure 4). DPFS was found to be 80% (SEM 0.103) after 60 months (Figure 5).

### Patterns of relapse

Seven patients (35%) developed relapsing disease after a median of 31 months (range 6 - 65 months). Four patients (20%) suffered from second relapses and three (15%) from third relapses after an additional median time of 22 and 3 months, respectively. Five of seven first relapses were local posterior fossa relapses, whereas two were spinal, one being limited to single segments of the thoracic spinal cord, another one extending from the skull base throughout the cervical spinal cord. In two out of five patients with locally recurrent disease, initial neurosurgery had not been complete (40%) as opposed to four incomplete resections in 13 recurrence-free patients (30.77%).

After diagnoses of first relapses, 2 patients underwent re-resection with adjuvant re-irradiation yielding an additional progression-free survival of 6 and 7 months, respectively. One patient was reirradiated only, and two were administered multiagent chemotherapy according to individual institutional protocols. In two patients, only palliative supportive treatment was performed.

Five patients died during follow-up-examinations (25%), including all four patients who had developed second relapses and two, in whom incomplete neurosurgical tumor resection was performed during initial treatment. Two patients with spinal recurrent disease were soon lost to follow-up, one after continuing therapy outside of Germany, the other one with extensive spinal disease rejecting further antitumor therapy.

### Prognostic factors

Metastatic disease at primary diagnosis did not predict for poor OS, local or distant PFS. Also, age (21 ≥ versus > 21, and 18 ≥ versus > 18), tumor localisation (medial versus lateral), administration of chemotherapy (yes versus no), neither combined nor adjuvant to radiation, and extent of resection (complete versus incomplete) did not significantly alter OS, local or distant PFS (table 1, additional file 1). In univariate analyses, OS was significantly decreased by the formation of local and spinal metastases after initial therapy (p = 0.02). Additionally, LPFS was significantly reduced in those patients developing spinal metastases (p = 0.01). Male sex predisposed for better local control (p = 0.04), possibly owing to only seven female patients included in this analysis, among whom two did not complete CSI and chemotherapy during first-line therapy. One had initially been misdiagnosed with cerebellar metastases of unknown origin, and another female patient had presented in very poor condition not eligible for CSI immobilisation.

**Table 1:** Non-significant prognostic factors

Non-prognostic factors	OS (*p*)	LPFS (*p*)	DPFS (*p*)
Primary metastatic disease	*0.36*	*0.35*	*0.53*
Infiltration of the fourth ventricle	*0.45*	*0.33*	*0.8*
Tumor location (lateral vs. medial)	*0.48*	*0.29*	*0.53*
Age (≤18 y vs. >18 y)	*0.22*	-	*0.11*
Age (≤21 y vs. >21 y)	*0.5*	*0.73*	*0.62*
Extent of resection (complete vs. partial)	*0.43*	*0.97*	*0.19*
Administration of chemotherapy	*0.7*	*0.59*	*0.27*

Statistical analysis revealed non-signficance for multiple prognostic factors on OS, LPFS, and DPFS.

We evaluated affection of the fourth ventricle in our cohort based upon preoperative MRI neuroimaging and surgical reports. Nine patients (45%) within our group showed histologically confirmed tumor cell invasion into the fourth ventricle or extensive distortion of the fourth ventricle at primary diagnosis, indicative of direct tumor infiltration. Patients with locally infiltrating tumors were less likely to undergo complete tumor resection (44.4% vs. 81.8%). Of those patients without tumor infiltration of the fourth ventricle, 82% lived after a median follow-up of 59 months, as opposed to 67% with initial tumor infiltration. However, OS, local PFS, and distant PFS were not significantly affected by the extent of ventricular infiltration (p > 0.05; table 1).

**Figure 6:**
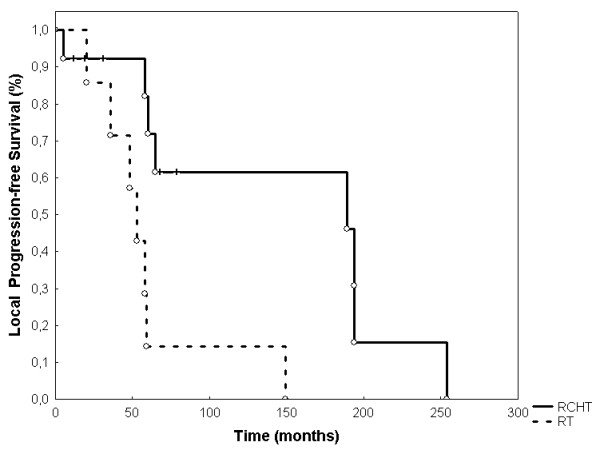
**Local progression-free survival with adjuvant radiotherapy versus radiochemotherapy (RCHT; concomitant and adjuvant chemotherapy)**. The addition of chemotherapy achieves favourable, but not yet significant results for local progression-free survival (p = 0.19).

In our cohort, 70% of all patients received chemotherapy, either prior to irradiation (5%), combined with radiotherapy (35%) or in an adjuvant setting (60%). All patients who had received combined radiochemotherapy, also received adjuvant chemotherapy. Six patients (30%) never entered any chemotherapy-containing therapy protocol. Although, addition of chemotherapy did not significantly influence OS as well as local and distant PFS, there seemed to be favorable results for those patients who were administered combined and adjuvant chemotherapy (p = 0.19; Figure 6).

All patients received RT after surgery. Most patients underwent CSI with additional boosts; however, two patients received whole brain irradiation with doses of 36 Gy and 40 Gy, respectively, followed by boosts to the posterior fossa with an additional 18 Gy and 8 Gy, respectively. One of these patients had initially been suspected to suffer from cerebellar metastases of unknown origin, and rejected the addition of spinal RT after histological confirmation of desmoplastic medulloblastoma. This patient developed spinal metastases within 42 months after initial treatment which required addition of local RT. The patient suffered from three further spinal relapses causing death after a total time period of 110 months. The other patient who received WBRT instead of CSI presented with very low performance status after tumor resection and was unable to tolerate the time necessary for CSI immobilization; this patient presented with local relapse 16 months after finishing whole brain irradiation and died shortly afterwards. Only in one patient, chemotherapy consisting of procarbazine, vincristine and methotrexate was administered before initiation of radiotherapy. This patient ultimately died from repeatedly relapsing disease 65, 101 and 124 months after primary diagnosis.

## Discussion

In the present analysis, we evaluated therapy response and outcome in 20 patients with desmoplastic medulloblastoma, who had received radiotherapy after tumor resection in combination with or without chemotherapy. We found high rates of EFS, OS, LPFS, and DPFS with moderate toxicity in the majority of patients. In our cohort, all patients received RT, but less than 50% were administered chemotherapy according to pediatric protocols, still showing quite favorable results when compared to other studies. Due to lack of standardized protocols for the treatment of adults with medulloblastomas, numerous individual treatment strategies for non-pediatric patients have evolved that abandon combined or adjuvant addition of chemotherapy and alter dosage and fractionation scheme of RT. Our patients with desmoplastic medulloblastoma turned out to profit most from postoperative CSI with combined and adjuvant chemotherapy and achieved good results also at tumor stages that have been shown to predispose for poor outcome in classical medulloblastoma. As opposed to those rare patients with primary metastatic disease or deep locoregional infiltration, in whom no significant reduction of OS, LPFS, and DPFS was detected, the formation of spinal metastases and locally recurrent disease after completion of any sole or combined therapy significantly decreased these patients' outcome. OS in relapsing disease was calculated to be 4 years, and not yet reached in those patients with controlled disease (p = 0.02).

In the era of risk adapted therapy approaches, predictors of good and bad therapy response must be clearly identified. Clinical parameters such as age, metastatic stage at primary diagnosis, or extent of initial surgery are useful for stratification of patients into low- and high-risk medulloblastoma therapy groups and have proven efficacy in children with medulloblastoma [21-23]. Subclinical parameters such as histological analyses have not yet been integrated into the process of therapy stratification in the management of medulloblastoma. However, in desmoplastic histology variant, which has repeatedly been suggested as a positive predictor, chromosome 9q loss of heterozygosity (LOH) is quite exclusively detected and cannot be found in classic histology, providing independent evidence that desmoplasia may indeed be pathogenetically distinct from its non-desmoplastic counterpart and therefore requires individual therapy regimens based upon individual patterns of risk factors [16,24].

The treatment of patients with medulloblastoma, especially in the adult population, is currently being evaluated in clinical trials. This directs attention towards desmoplastic histology, as it is a more frequent finding in adult patients. In the present analysis of patients with desmoplastic histology, most patients were adults, thus displaying an appropriate cohort for future studies on non-pediatric medulloblastoma patients who may not have been sufficiently represented and analysed in former pediatric studies. Patients with desmoplastic histology have been discussed controversially with respect to outcome compared to patients with classical medulloblastomas or supratentorial PNET [3,25-27]. Rutkowski et al. have demonstrated desmoplastic histology to be an independent and significant prognostic factor for favorable outcome in young children compared with classic histology (10 years PFS 85% versus 34%) [16].

Several authors have looked for general differences between pediatric and adult desmoplastic medulloblastoma, and have found that despite differing growth rates, which they see as age-related factors, there are major similarities concerning typical biochemical and histological features of desmoplasia [11,28]. Independent of patient age, probes are immunoreactive with markers of neuronal commitment such as tubulines and neurofilaments; a more mature neuronal character in adult desmoplastic medulloblastoma was detected [29]. However, since there are major clinical differences between adult and pediatric patients concerning treatment-related side effects, such as radiation-associated mental impairment, a clear distiction must be made between these two groups. Recent studies have proven efficacy of intensive chemotherapies in young children under the age of three, in whom CSI can possibly be avoided or at least delayed in order to prevent neurological deficits [30]. There were no children under the age of three in this group.

Outcome observed in this analysis of mostly adult patients compares well to published results on treatment of both medulloblastoma in general and desmoplastic medulloblastoma. OS rates for patients within multimodal therapy regimens have previously been reported to range from 50% to 70% at 60 months, and from 30% to 50% at 120 months [8,31,32]. In our cohort, OS at 60 months was 80%, and 54.9% at 120 months. In a retrospective analysis of 66 patients performed at the Department of Radiooncology at the University Hospital of Heidelberg, desmoplastic medulloblastoma patients fare better than their classic counterparts, though statistical significance was not yet reached due to small patient numbers (OS at 5 years: 80 vs. 68%, p = 0.097) [33]. Similar to adult medulloblastoma in general, where 5 years of local progression-free survival are reported in 67% [34], posterior fossa control rates in our group of mostly adult desmoplastic medulloblastoma reached 71.2%.

Desmoplastic histology may have been underestimated as a predictor of good response due to its frequent finding in non-pediatric patients, in whom adjuvant chemotherapy is not as routinely administered as in children despite possible contributions to better outcome; however, it can be argued that addition of chemotherapy may offer significant benefit also in the adult population [35]. Due to the lack of standardized treatments protocols for adult patients, chemotherapy is not administered on a general basis in the adult population. Combined and adjuvant chemotherapy was administered in only 7 patients (35%) in the group reported here. Treatment protocols evaluating the addition of combined and adjuvant vincristine-based chemotherapy to CSI have led to the conclusion that a reduction in CSI doses from 35.2 to 23.4 Gy does not decrease survival rates. However, lower doses of RT can help prevent long-term treatment-associated adverse events [36,37]. In our group of patients, both acute and chronic therapy-related toxicity was moderate, even though CSI doses were reduced to 23.4 Gy in only four patients (20%), all of whom were subjected to chemotherapy-containing regimens. However, all patients who suffered from chronic CNS toxicity such as pituitary gland failure or cognitive impairment had received CSI doses of 36.0 Gy with additional boosts of 54.0 Gy and without chemotherapy. On the contrary to radiogenic side effects, chemotherapy-associated toxicity such as acute nausea, and chronic peripheral neuropathy was documented in up to 42% of the patients treated. Limited irradiation of involved CNS areas may be a future option in pediatric patients, but cannot be recommended in adult patients. In our cohort, two adult patients did not receive CSI - one due to misdiagnosis, the other one due to low performance status. Both patients suffered from early tumor relapses.

One secondary malignancy occurred seven years after treatment and was an osteosarcoma of the skull base in one pediatric male patient (5%). This tumor was treated successfully with surgical resection and additional RT. According to previous data, malignant bone tumors after radiotherapy for medulloblastoma must be expected in 7.4% of all patients [38], which emphasizes the need for continuous follow-up examinations and employment of less toxic regimens. In our case, the patient had received a CSI dose of 35.2 Gy, a boost of 55 Gy in combination with concomitant and adjuvant chemotherapy.

Administration of chemotherapy has been shown to improve OS, LPFS and DPFS. In our cohort, addition of chemotherapy did not influence therapy outcome significantly, which however may be due to the small number of patients (n = 14), in whom chemotherapy was administered [39]. Also, lack of significance may be attributed to the heterogeneity of chemotherapies used. However, we did observe a trend towards better outcome in those seven patients who received radiation therapy in combination with weekly infusions of vincristine and several adjuvant cycles of vincristine, lomustine and carboplatin. None suffered from recurrent disease, and LPFS appeared superior to regimens without chemotherapy, though statistical significance was not yet reached. Supporting the need for chemotherapy, none of five patients dying from medulloblastoma in this cohort had received combined adjuvant radiochemotherapy (additional file 1).

In some reports, infiltration of the fourth ventricle, especially of its floor, has been shown to possibly enhance the risk of tumor dissemination. This is mainly due to less probable complete resection and closer proximity to spinal fluid circulation and, thus, imposes a greater risk of distant and posterior fossa recurrence and accounts for poor outcome [3,9]. However, the statistical significance of locoregional tumor infiltration as predictor of poor outcome remains controversial [9,39], and our analysis did not detect any significant impact on therapy response.

In several previous studies, both the initial spread and dissemination of disease and postoperative tumor residues significantly determined outcome and prognosis in patients with classic medulloblastoma [17,25,34]. In contrast, in our group of patients with desmoplastic medulloblastoma, outcome was influenced by the success of combined first line therapies rather than the initial stage of disease or extent of surgery. All patients with relapsing tumor either died from medulloblastoma or were soon lost to further follow-up, most likely indicating progression to death. On the contrary, initial locoregional infiltrative and metastatic disease or postoperative residual tumor did not significantly influence outcome. This finding, however, is limited by the fact that only 50% of the patients received postoperative cross-sectional imaging, with 40% of them being subjected to CT scans instead of MRI scan analysis, which is now known to provide more reliable information about the extent of resection. Being a rare diagnosis, the identification of prognostic factors and their statistical evaluation is compromised by the small number of patients.

## Conclusions

Tumor features at initial diagnosis such as lateral versus medial location, infiltration of the fourth ventricle, metastatic disease, extent of resection or age did not affect therapy outcome in our cohort. This supports the inclusion of patients with advanced disease in extensive treatment protocols, still with curative intent. However, treatment failure after first line regimens, such as spinal recurrence of disease significantly impaired outcome. Recurrence of disease occurred as late as 65 months after completion of initial therapy, thus emphasizing the need for continuous follow-up examinations over many years. Radical surgery to a reasonable extent followed by radiotherapy and both combined and adjuvant chemotherapy according to pediatric protocols provide the best results to date and are confirmed by our data. We collected our data retrospectively and not from a controlled investigator-blinded clinical study. Therefore, future prospective studies are needed to confirm the most effective combination of treatment modalities and to clarify the role of desmoplastic histology as a prognostic factor itself.

## Competing interests

The authors declare that they have no competing interests.

## Authors' contributions

SR collected the patients' data, performed all statistical analyses and wrote the manuscript. TG performed the neuropathohistological analyses. AM made substantial contributions to collecting the patients' data. TW analysed CT- and MR-images of all medulloblastoma patients. OW, AK, and WW supervised zytostatic treatment, documented side effects and organized post-treatment follow-up examinations. JD planned and supervised radiation treatment. SC conceived of the study and helped to write and finalized the manuscript. All authors helped with the interpretation of the data, read and approved the final manuscript.

## Pre-publication history

The pre-publication history for this paper can be accessed here:

http://www.biomedcentral.com/1471-2407/10/450/prepub

## Supplementary Material

Additional file 1 **Patient characteristics**. individual characteristics of 20 patients with histologically confirmed desmoplastic medulloblastoma. Click here for file
