# Supplementary material for: Outcome and prognostic factors of desmoplastic medulloblastoma treated within a multidisciplinary treatment concept
Source: BMC Cancer. 2010 Aug 23;10:450. doi: 10.1186/1471-2407-10-450 (PMC2939548; doi:10.1186/1471-2407-10-450)
Supplement: Additional file 1 — Patient characteristics. individual characteristics of 20 patients with histologically confirmed desmoplastic medulloblastoma. [file 1471-2407-10-450-S1.DOC]

**Suppl. Table**

| Patient no. | OS | LPFS | DPFS | metastases at first diagnosis | cerebellar localization | fourth ventricle | state of resection | adjuvant RCHT | age at first diagnosis | last follow-up |
| --- | --- | --- | --- | --- | --- | --- | --- | --- | --- | --- |
|  | *months* | | |  |  |  |  |  | *years* |  |
| 1 | 6 | 5 | 5 | yes | lateral | infiltrated | incomplete | no | 8 | alive |
| 2 | 20 | 20 | 20 | no | lateral | not infiltrated | complete | yes | 35 | alive |
| 3 | 22 | 19 | 22 | no | medial | infiltrated | incomplete | no | 21 | deceased |
| 4 | 25 | 12 | 25 | no | lateral | infiltrated | complete | no | 25 | deceased |
| 5 | 36 | 36 | 36 | no | lateral | not infiltrated | complete | yes | 36 | alive |
| 6 | 48 | 48 | 48 | no | lateral | not infiltrated | complete | yes | 16 | alive |
| 7 | 53 | 53 | 53 | no | lateral | infiltrated | complete | yes | 30 | alive |
| 8 | 58 | 58 | 58 | no | lateral | not infiltrated | complete | no | 37 | alive |
| 9 | 58 | 58 | 58 | no | lateral | not infiltrated | complete | yes | 23 | alive |
| 10 | 59 | 59 | 59 | no | lateral | not infiltrated | incomplete | yes | 12 | alive |
| 11 | 60 | 60 | 42 | yes | medial | infiltrated | complete | no | 21 | alive |
| 12 | 60 | 31 | 40 | no | lateral | not infiltrated | complete | no | 41 | deceased |
| 13 | 65 | 65 | 65 | yes | lateral | infiltrated | complete | no | 38 | alive |
| 14 | 110 | 79 | 43 | no | lateral | not infiltrated | complete | no | 50 | deceased |
| 15 | 149 | 149 | 149 | yes | lateral | infiltrated | incomplete | yes | 7 | alive |
| 16 | 152 | 68 | 152 | no | medial | infiltrated | incomplete | no | 3 | deceased |
| 17 | 189 | 189 | 189 | no | lateral | not infiltrated | complete | no | 26 | alive |
| 18 | 194 | 194 | 194 | no | lateral | not infiltrated | incomplete | no | 32 | alive |
| 19 | 194 | 194 | 194 | no | medial | infiltrated | incomplete | no | 11 | alive |
| 20 | 254 | 254 | 254 | no | lateral | not infiltrated | complete | no | 11 | alive |
